# Radiation Resistance: A Matter of Transcription Factors

**DOI:** 10.3389/fonc.2021.662840

**Published:** 2021-06-01

**Authors:** Chiara Galeaz, Cristina Totis, Alessandra Bisio

**Affiliations:** Laboratory of Radiobiology, Department of Cellular, Computational and Integrative Biology (CIBIO), University of Trento, Trento, Italy

**Keywords:** radiotherapy, transcription factors, radiation resistance, cancer stem cells, ROS - reactive oxygen species, inflammation

## Abstract

Currently, radiation therapy is one of the standard therapies for cancer treatment. Since the first applications, the field of radiotherapy has constantly improved, both in imaging technologies and from a dose-painting point of view. Despite this, the mechanisms of resistance are still a great problem to overcome. Therefore, a more detailed understanding of these molecular mechanisms will allow researchers to develop new therapeutic strategies to eradicate cancer effectively. This review focuses on different transcription factors activated in response to radiotherapy and, unfortunately, involved in cancer cells’ survival. In particular, ionizing radiations trigger the activation of the immune modulators STAT3 and NF-κB, which contribute to the development of radiation resistance through the up-regulation of anti-apoptotic genes, the promotion of proliferation, the alteration of the cell cycle, and the induction of genes responsible for the Epithelial to Mesenchymal Transition (EMT). Moreover, the ROS-dependent damaging effects of radiation therapy are hampered by the induction of antioxidant enzymes by NF-κB, NRF2, and HIF-1. This protective process results in a reduced effectiveness of the treatment, whose mechanism of action relies mainly on the generation of free oxygen radicals. Furthermore, the previously mentioned transcription factors are also involved in the maintenance of stemness in Cancer Stem Cells (CSCs), a subset of tumor cells that are intrinsically resistant to anti-cancer therapies. Therefore, combining standard treatments with new therapeutic strategies targeted against these transcription factors may be a promising opportunity to avoid resistance and thus tumor relapse.

## Introduction

Since the beginning of the last century, radiotherapy, together with surgery and chemotherapy, has played a crucial role in cancer treatment. The efficacy of radiotherapy, as a curative and palliative strategy, promoted technical advances in this field, in order to deliver higher doses to the tumor in a more precise way, sparing healthy tissues and reducing side effects (1). This resulted in a significant number of patients (estimated to be around 50%) that currently undergo external beam radiotherapy during their treatment (2). The most common treatment regimen consists in a standard fractionated irradiation, usually of 1.8-2 Gy per day, 5 days a week, to limit side effects to normal tissues (1, 3). Some exceptions exist in conditions in which it is possible to deliver a higher dose in a radiation field that is confined to the tumor tissue, such as for brain metastases (4). Additionally, there is accumulating evidence in support of the use of hypofractionation for different cancer types, as for prostate cancer, in which an increase in the dose per fraction is not associated with a higher late toxicity (5). Hypofractionation can be generally divided in moderate, consisting in 2.4-3.4 Gy per fraction, or ultrahypofractionation, where the dose reaches 5 Gy per fraction. These more recent clinically used fractionation protocols present the advantage of reducing the number of fractions and the treatment time for the patient, being considerably more cost-effective compared to the conventional approaches.

Generally, the fractionation permits: i) the reoxygenation of the tumor during the treatment period, ii) the redistribution of cells in cell cycle phases in which they are more susceptible to irradiation, iii) the impairment of repopulation of the tumor mass, as reviewed by Huber and colleagues (6), presenting several advantages over the single-dose exposure.

Nevertheless, the main limitation of this treatment strategy is the possibility of some cells to develop mechanisms of resistance during consecutive exposures to ionizing radiations (IR) (7, 8). Radiosensitivity is considered the 5^th^ R of radiotherapy (9) in addition to the four proposed by Withers (10), which represent different cellular aspects that affect the efficacy of fractionated radiotherapy and may explain the sparing of normal tissues. The effectiveness of IR in the complete eradication of the malignant mass can change depending on the mechanisms that are already activated in cancer cells or the ones that can be induced following irradiation. This difference sets the basis to distinguish resistance to radiotherapy in two main categories: intrinsic and acquired. The former is present in cancer cells even before the starting of the treatment, and it relies on some inherent characteristics of the tumor (11). In particular, the presence of cancer stem cells (CSCs) in the tumor bulk plays a prominent role in this type of cancer radioresistance (12–14). CSCs are a small subset of cancer cells that is composed of a reservoir of self-sustaining cells with the unique potential to self-renew and maintain the tumor (15). If the radiation dose administered is not sufficient to cause a significant reduction in the CSC population, the frequency of those stem cells could increase during treatment, even if, macroscopically, the tumor regresses. This enrichment in CSCs number after conventional radiotherapy has been demonstrated in lymphoma, breast, and hepatocellular carcinoma cell lines (16) and also confirmed recently in prostate cancer (17).

On the other side, acquired radioresistance is a process of adaptation of cancer cells to the changes induced by irradiation itself, which finally results in resistance to the treatment (18). This can be achieved through some transcription factors (TFs) whose activation following radiation permits the cancer cells to escape the lethal effects of IR.

In this review, we will focus on the main TFs which are activated in irradiated cancer cells, trying to draw attention to the downstream mechanisms through which they induce the survival of malignant cells.

Several reports highlight that Signals Transducers and Activators of Transcription 3 (STAT3) is involved in cancer radioresistance. In the late ‘90s, two independent studies described the existence of the third member of the STAT family of proteins. In the first one, Akira and colleagues (19) identified a new acute-phase response factor (named APRF at the time) with more than 50% homology with p91 (also known as STAT1) but responding to different stimuli (Interleukin-6, IL-6, and Oncostatin M instead of the canonical Interferon-gamma). On the other hand, the discovery of a protein activated by Epidermal Growth Factor (EGF) and IL-6 and able to form heterodimers with STAT1, gave support to the finding of this novel factor (20).

Nowadays, STAT3 is a well-known transcription activator of 92 kDa encoded on the chromosome 17q21.2. The protein is composed of a coiled-coiled domain at the N-terminus, followed by a DNA binding domain, a linker sequence, which connects the latter with the SH2 domain and, finally, the transactivation domain in the C-terminus (21). In this terminal part, there are two specific amino acids, a tyrosine and a serine, in position 705 and 727, respectively, which are extremely important for the activation of the protein. Indeed, as it is summarized by Aggarwal and colleagues (22), the phosphorylation of these two residues is necessary for the activation of STAT3, which can subsequently homo- or hetero-dimerize and translocate into the nucleus, where it carries out its role as a transcription activator.

The stimulation of STAT3 by cytokines evidences a link between inflammatory pathways and radioresistance, also highlighted by others (23). This observation can be further supported by the involvement of another inflammation-related TF in tumor radioresistance: Nuclear Factor kappa light chain enhancer of activated B cells (NF-κB).

The Nuclear Factor-kappa B (NF-κB)/Rel proteins are a family of transcription factors involved in different biological processes, such as immune system development, inflammation, cellular growth, and apoptosis (24, 25). There are several pieces of evidence that, in addition to these physiological roles, indicates NF-κB proteins are involved in the development of malignancies and thus contribute to cancer progression (26). This family is composed of 5 members which form homo- or hetero-dimers: RelA (p65), RelB, cRel, NFKB1 (p50/p105) and NFKB2 (p52/p100). All these components are characterized by the presence of a REL Homology Domain (RHD), which allows the interaction with DNA at the κB sites. In normal conditions, the NF-κB complexes are inactive and therefore retained in the cytoplasm by IκB proteins, the negative regulators of NF-κB. These inhibitory proteins mask the NLS (Nuclear Localization Signal) present in NF-κB proteins, impeding their nuclear translocation (27). After specific stimuli, the IκB Kinase (IKK) complex, formed by two kinases (IKKα and IKKβ) and a regulatory subunit (IKKγ/NEMO), is activated and it is responsible for the phosphorylation of two residues at the N-terminus of IκB: serine 32 and serine 36 (28). These modifications promote the ubiquitination of IκB and its subsequent degradation by the proteasome, allowing the nuclear translocation of NF-κB complexes, where they can exert their transcriptional functions. Moreover, NF-κB factors can also be activated by a non-canonical pathway, which is involved in the non-inflammatory signaling. This alternative pathway requires the activation of NF-κB-Inducing Kinase (NIK), which phosphorylates p100 and recruits IKK protein. The phosphorylation of p100 by IKK stimulates its partial proteolytic degradation to the p52 form, which, once free from its inhibitory region, is able to dimerize with RelB and move into the nucleus to promote transcription of target genes (29). Constitutive activation of NF-κB is frequently observed in different types of human cancer (30), therefore a complete understanding of its role in the growth of malignancies and in the development of treatment resistance is needed.

Even if inflammatory TFs play a crucial role in radioresistance, they are not the only factors that are activated in cancer cells to avoid apoptosis. It is widely recognized that radiation treatment causes damages to cancer cells through a direct and an indirect way (31). The direct mechanism of action relies on the effects of ionizing photons and particles which cause macromolecule alterations, while the indirect DNA damage is mainly due to the production of free radicals from ionized water molecules. The production of reactive oxygen species (ROS) and the subsequent DNA damage are considered the main processes through which cancer cells are killed by IR (32). This implies that the increase in ROS concentration in the tumor microenvironment and in the cells is the starting point for the induction of effective antioxidant responses in malignant cells in order to survive to stressful conditions (33, 34). Nuclear factor (erythroid-derived-2)-like 2 (NRF2) is a key TF which regulates the expression of a variety of genes involved in the maintenance of redox homeostasis and in the protection against oxidative stress. In normal conditions, it is sequestered in the cytoplasm by Kelch-like ECH-associated protein 1 (KEAP1) and degraded in a ubiquitin/proteasome-dependent manner. During oxidative stress, modifications of KEAP1 cysteines lead to conformational changes in the enzyme and subsequent dissociation of NRF2 from KEAP1 (35, 36). This results in the nuclear translocation of the TF, where it heterodimerizes with small Musculoaponeurotic fibrosarcoma proteins (sMafs), binding to antioxidant response elements (AREs) in the regulatory regions of its target genes (37).

For a long time, NRF2 has been considered a cytoprotective TF, due to its role as a defense mechanism of the cells, and a tumor suppressor gene for its function in chemoprevention. However, different studies have highlighted the enigmatic findings regarding the role of NRF2 as an oncogene or a tumor suppressor (37, 38). In addition, this TF is associated with radioresistance in different malignancies, and its crosstalk with other genes related to resistance to IR (NF-κB, Hypoxia Inducible Factor-1, HIF-1, p21) render it a promising target to overcome treatment limitations (32).

Soon after the broad application of IR in cancer treatment, the importance of oxygen and its association with radiosensitivity was realized (1). Indeed, it is known that a characteristic of solid tumors is to present a hypoxic environment that seems to be highly correlated with an increased resistance to chemotherapy and radiotherapy (39). The leading player acting in conditions of low oxygen concentration is HIF-1, which is composed of two subunits: HIF-1α and HIF-1β. The latter is constitutively expressed, while the activity of HIF-1α is dependent on oxygen levels, therefore determining the activation of the HIF-1 heterodimer only in hypoxia. Indeed, in normoxia, HIF-1α is hydroxylated at two proline residues (Pro 402 and Pro 564) by Prolyl-4-hydroxylases (PHDs) (40). These modifications are responsible for the recruitment of the tumor suppressor von Hippel-Lindau (VHL), which is promoting α-subunit poly-ubiquitination and degradation by the proteasome (41). Since PHDs need oxygen to work properly, during hypoxic conditions HIF-1α is no longer modified by these enzymes. Therefore, the recruitment of VHL is abolished and HIF-1α is free to translocate into the nucleus where, together with HIF-1β, forms a stabilized HIF-1 heterodimer. This complex then binds the DNA at Hypoxia Responsive Elements (HREs) that are found in specific promoters or enhancers, leading to the transcription of the correlated genes, which are associated with different cellular functions.

Radioresistance leads to cancer recurrence, metastasis, and poor survival of cancer patients; for this reason, understanding the molecular mechanisms that are activated in cancer cells and which underlie both intrinsic and acquired radioresistance is extremely important to develop effective combination treatments to overcome the malignancy permanently.

In this review, we will focus mainly on the role of the TFs previously mentioned and the pathways which are activated downstream, finally leading to the acquisition of resistance to IR in various cancer types. Moreover, we will consider some recent findings regarding the most promising inhibitors of these TFs. In our opinion, this will help decipher innovative combinatorial strategies involving radiotherapy and inhibitors of these TFs, which in the future could have a critical role in overcoming radioresistance. Due to the paucity of studies regarding charged particle therapy, we will mostly take into account results regarding conventional radiotherapy with X-rays, with some hints to recent discoveries on proton and carbon ion-induced resistance.

## Cancer Stem Cells (CSCs)

Experimental evidence has accumulated proving that, to avoid tumor recurrence, all CSCs should be inactivated by the treatment. Indeed, as outlined above, CSCs are thought to be the unique subset of cells in a tumor which retain a tumorigenic potential and, therefore, lead to tumor relapse. The major problem linked to current therapies is the fact that they are optimized for decreasing the tumor bulk and, consequently, they mainly act on non-stem cancer cells, leaving CSCs untouched. Moreover, CSCs have been demonstrated to be particularly resistant to cancer treatments, owing to different intrinsic characteristics typical of these cells. For example, for what concerns chemotherapy, CSCs showed to be less sensitive to certain drugs due to the increased expression of different ATP-Binding Cassette (ABC) transporters, which actively pump out the toxic molecules from the cell, strongly reducing the treatment effectiveness (42). In addition, these tumorigenic cells present a higher DNA repair ability if compared with classic cancer cells, thus they are intrinsically resistant to radiotherapy, whose mechanism of action depends on DSBs induced by the generation of ROS. This mechanism of resistance seems to be principally linked to the activation of two important kinases: ATM and ATR. Their signaling pathways, in particular the downstream kinases CHK1 and CHK2, set up the conditions to allow DNA repair in cancer cells, protecting them from cell death (43, 44). Another advantage that leads to radioresistance in CSCs is the fact that they exhibit low ROS levels, due to an increased expression of ROS scavenging molecules, such as GSH, SOD, Catalase, and Thioredoxin (45). This characteristic is exploited by stem-like cancer cells to cope with oxidative stress generated by ionizing radiation, minimizing the effects of the treatment. Moreover, the intrinsic resistance shown by CSCs is also related to their ability to enter a quiescent state, remaining in the G0 phase for long periods as “dormant” CSCs (46). Given the DNA-damaging therapies rely on cell growth, this state of reduced proliferation allows CSCs to survive anticancer treatments. Eventually, a relevant role in the resistance of CSCs is played by specific areas present within the tumor mass, where stem-like cells are thought to be located: the niches. Here, CSCs can find the right conditions to be protected from different stress-inducing agents, therefore surviving radiation treatments and causing tumor relapse.

CSCs have an important role also in acquired resistance, which develops as a result of cancer therapies and has been associated with different biological processes. Indeed, surviving CSCs, through asymmetric division stimulated by anticancer treatments, contribute to cell heterogeneity within the tumor and increase the abundance of the CSC population (8). In addition, the tumor microenvironment (TME) plays an important role in the acquisition of radioresistance. As a matter of fact, some immune cells and Cancer-Associated Fibroblasts (CAFs) contribute to the development of a resistant phenotype through the production of several soluble factors, such as cytokines, chemokines, and growth factors (46), which stimulate molecular mechanisms involved in the development of a radioresistant phenotype. Furthermore, radioresistance acquisition in non-CSCs is promoted by the activation of pro-survival signaling pathways, such as AKT and mTOR. Indeed, Shimura and co-authors (47) showed that fractionated radiotherapy induced the over-expression of Cyclin D1, as a result of constitutive DNA-PK and AKT activation with concomitant reduction in glycogen synthase kinase 3β (GSK3β)-mediated Cyclin D1 degradation. This mechanism has been demonstrated to be fueled by a positive feedback loop, since Cyclin D1 over-expression leads to a constitutive activation of the DNA damage response. The radioresistant phenotype has also been associated with the activation of mTOR pathway by Chang and colleagues (48), which observed a mTOR-dependent appearance of mesenchymal and CSC phenotypic traits after repeated irradiation. In addition, Chen and collaborators (49) demonstrated that the inhibition of mTOR with rapamycin hampered DSB-repair pathways. In conclusion, it can be inferred that AKT and mTOR pathways are involved in radioresistance acquisition, mainly by increasing the DNA-repair ability of cancer cells.

CSCs are characterized by specific surface markers, depending on the type of cancer they belong to, and, generally, high expression of these markers in a tumor is correlated with an adverse clinical outcome. For example, an established marker of CSCs in brain tumors is CD133, a five-transmembrane glycoprotein with unknown functions (50). Particularly, two CD133 glycosylated epitopes, AC133 and AC141, seem to be enriched in the highly tumorigenic subpopulation of brain tumor stem cells (51). In breast cancer, instead, a subpopulation of CD24^-/low^/CD44^+^ cells has been identified to include tumor-initiating cells and it has been demonstrated that as few as 100 of these cells were able to form tumors in mice (52). Moreover, these Breast Cancer Stem Cells (BCSCs) present an increased capability of DNA repair and an efficient ROS scavenging system, two characteristics that make them highly radioresistant (53). Furthermore, these CSCs were also found to be positive for ALdehyde DeHydrogenase 1 (ALDH1) and presented a reduced 26S proteasome activity. This last characteristic is not only typical of BCSCs, but it is frequently found in CSCs of other tumor types, such as glioma, Non-Small Cell Lung Carcinoma (NSCLC), Head and Neck Squamous Cell Carcinoma (HNSCC) (54–56). Also, the ALDH1 positivity is not an exclusive of BCSCs, but it is typical of CSCs from other tumors, like colorectal cancers and gliomas (13).

It is well-established that stemness is governed by several transcription factors and that their silencing causes cell differentiation. The most relevant factors involved in self-renewal and plasticity are the four so-called “Yamanaka” factors: SOX2, OCT4, KLF4, and c-MYC (57). These genes are necessary and sufficient to induce pluripotency in somatic cells. Of particular relevance, also NANOG, another stemness-related gene, can substitute, together with Lin28, c-MYC and KLF4 in the induced Pluripotent Stem Cells (iPSCs) protocol (58). This transcription factor is frequently found up-regulated in CSCs and is crucial for the self-renewal of stem-like cells. It is remarkable that the Yamanaka factors and NANOG are all degraded by the 26S proteasome, whose activity is reduced or absent in CSCs, suggesting a functional role of this characteristic commonly presented by CSCs (16). In addition to these canonical genes, which are strictly correlated with stem-like properties, there are also other transcription factors which have shown to be important in the maintenance of the undifferentiated state in CSCs. The first example is represented by HIF-1, which is activated in hypoxia, a condition that can be found in niches where CSCs reside. Indeed, the hypoxic environment can be exploited by CSCs to better cope with oxidative stress-inducing treatments, like radiotherapy, thus preventing adequate tumor elimination. This ability conferred by HIF-1 is due to the fact that the transcription factor is inducing the expression of several genes that help the cells to deal with ROS exposure, such as GSH, as previously mentioned. Furthermore, HIF-1 seems to have a critical role in the preservation of the undifferentiated state of cancer cells. Indeed, this hypoxia-related TF positively regulates several stemness-related genes mentioned above, such as OCT4, NANOG, SOX2, KLF4, and NOTCH (59, 60). Moreover, HIF-1 activity leads to the activation of WNT-Catenin signaling pathway, which confers stem-like properties to cancer cells and improves the DNA repair potential of these tumorigenic cells (61). CSCs display an up-regulation of another transcription factor critically involved in radioresistance: NF-κB (62, 63). Due to the implication of this family of proteins in maintaining a correct balance of ROS levels, it is thought that NF-κB owns an essential function in CSCs. Indeed, it regulates an important antioxidant gene, SOD2, which seems to be implicated in maintaining low levels of oxidative stress in CSCs, thanks to its scavenger role (64). Moreover, Qin and colleagues demonstrated that, in ovarian cancer stem cells, the hypoxic environment can activate NF-κB, together with HIF-1 (65). These two transcription factors in turn induce the expression of Sirtuin-1 (SIRT1), a gene with a critical role in tumorigenesis which positively regulates HIF-1 (65). This Sirtuins family member, a histone deacetylase, has shown to be involved in stemness, strongly influencing the expression of CSC markers and resistance to therapies. In addition to this, NF-κB, as well as STAT3, positively regulates the expression of Survivin, an anti-apoptotic gene involved in enhanced DNA repair activity (66), a typical characteristic of CSCs. In addition, STAT3 is believed to play an essential role in CSCs. Indeed, as noted above, it transcriptionally up-regulates SLUG, which in turn fosters radioresistance by promoting the expression of stem-like characteristics and anti-apoptotic proteins. Furthermore, the increase in ALDH expression has been demonstrated to be a consequence of STAT3 activity, specifically in triple-negative breast cancer as outlined by Arnold and collaborators (67). It is noteworthy to mention that STAT3 seems to be involved in a gain of cell plasticity after cell irradiation, sustaining an emerging hypothesis which supports the idea that IR might enhance CSC subpopulation (65, 68). Therefore, targeting STAT3 could be a decisive factor in determining an increased effectiveness of the treatment. Eventually, another transcription factor correlated with the maintenance of the undifferentiated state of CSCs is NRF2, whose knock-down not only significantly reduces stem cell markers in glioma stem-like cells, but also induces their differentiation, as demonstrated by Zhu and colleagues (69). Clearly, the contribution given by NRF2 in the maintenance of the stem cell characteristics and the low status of differentiation is linked to its ROS-modulating ability. Indeed, this transcriptional regulator promotes the expression of a wide range of antioxidant enzymes, such as HO-1, SODs, and NQO-1. Lastly, among the NRF2 target genes it is relevant to mention also drug efflux transporters (70), which is another feature that determines chemoresistance and it is frequently found in CSCs.

## Signals Transducers and Activators of Transcription 3 (STAT3)

### Mechanisms of STAT3 Activation

Initially considered as one of the key mediators of inflammation due to its activation by inflammatory cytokines, new and critical roles of STAT3 have been highlighted. Given its ability to transform cells when constitutively activated and due to its over-expression in a wide range of human cancers, STAT3 is currently considered as an oncogene. In this context, STAT3 plays a plethora of functions associated with tumorigenesis: i) it is able to induce the expression of different anti-apoptotic genes, suppressing the apoptosis of transformed cells; ii) it can lead to cellular proliferation through the expression of cyclins and other growth-associated oncogenes; iii) it stimulates cellular invasion, angiogenesis, and metastasis with the induction of the transcription of some mediators of these processes (22).

Moreover, some years ago, it has been proposed that STAT3 can also be involved in the resistance of cancer cells to IR. As a matter of fact, B-Cell Receptor (BCR) engagement in the presence of IL-6/IL-10 protects peritoneal B lymphocytes (also named B-1 cells) in mice treated with gamma radiation from radiation-induced apoptosis *via* activation of STAT3 (71).

The implication of STAT3 in the acquired radioresistance of cancer cells has been further demonstrated by several studies that analyzed the relationship between STAT3 and the ability of tumors to regrow after the treatment with IR. In particular, different reports have provided proofs of a correlation between the staining for STAT3 in human tumor specimens and the recurrence of glioblastoma multiforme (GBM) (72, 73). Similarly, the activation of STAT3 seems to be correlated with the relapse of patients after radiotherapy in breast cancer (74). The outcome also applies for pharyngeal cancers, in which the staining for phosphorylated STAT3 is significantly linked with the reduced complete response rate to definite chemotherapy and radiotherapy (75).

It is well known that various stimuli such as growth factors, cytokines, and oncogenic proteins can induce STAT3, so it is not surprising that this TF can be differently activated in response to the treatment of cancer cells with IR. Kim and collaborators showed that the expression of the Human Epidermal growth factor Receptor 2 (HER2) is associated with an higher resistance to radiotherapy in HER2-positive breast cancers compared to other molecular subtypes of breast cancer (74). This effect is due to the enhanced activation of STAT3 by HER2 after radiation, which promotes the transcription of downstream genes, including Survivin. As a consequence, the cells are protected from IR through the inhibition of apoptosis, the promotion of mitosis, and the enhancement of DNA repair.

Another common type of activation of the STAT3 signaling pathway is through the binding of some cytokines to their receptor. It has been demonstrated that the expression of IL-6 and its receptor (IL-6R) in tissue specimens and cancer cell lines of pharyngeal cancer is higher than in non-malignant tissues and the stimulation with the same cytokine or the blockade of IL-6R led to attenuation or augmentation of cellular death following radiation, respectively (75). The binding of IL-6 to the membrane receptor triggers the gp130 signal transducer and the subsequent activation of the tyrosine kinase Janus Kinase (JAK) and the tyrosine phosphorylation of gp130, subsequently activating the JAK/STAT pathway, which permits the phosphorylation and activation of STAT3. Once STAT3 is phosphorylated, it can form homo- or hetero-dimers (with STAT1 or p65), which can translocate into the nucleus to exert their function. The activation of this pathway is supposed to be responsible for a more aggressive tumor growth and resistance to radiation therapy in pharyngeal cancer (75). A similar mechanism confers radioresistance also to hepatocellular carcinoma (HCC): the inhibition of IL-6 signaling increases the radiotherapy-induced cell death, and this is associated with decreased phosphorylation of STAT3 (76). In prostate cancer, the up-regulation of IL-6 induced by irradiation on one side activates STAT3 signaling, while on the other induces androgen receptor expression. Moreover, in this tumor type, IL-6 silencing leads to the sensitization of tumor cells to irradiation through the increase of cell death and DNA damage (77). Considering the results of these studies together, it is remarkable the relevant role of cytokines, in particular of IL-6, in the activation of STAT3 signaling pathway, which finally allows cancer cells to become more resistant to IR. Such a system seems to be conserved in different cancer types, suggesting that inhibiting the signaling at various steps could be relevant to increase the sensitivity of cancer cells to radiotherapy.

Usually, most of the studies focused on the phosphorylation at tyrosine 705 as the main mechanism for the activation of STAT3. However, recently, the role of phosphorylated serine 727 has emerged as a key component in radioresistance of cancer cells. Ouedraogo and colleagues (78) highlighted that the accumulation of phosphorylated STAT3 on serine 727 is correlated with intrinsic radioresistance of some GBM cell lines and the decrease in this post-translational modification resulted in a significant radiosensitization. This observation needs further investigations to assess its importance in resistance to radiotherapy in other tumor types. Nevertheless, the phosphorylation on this second residue seems to be independent of the gp130/JAK activation, adding a new level of complexity to the STAT3-mediated radioresistance.

More recently, other mechanisms of STAT3 activation have been proposed. In HCC, the phosphorylation and activation of JAK2, which is mainly responsible for the phosphorylation of STAT3, appeared to be stimulated by Mucin 1 (MUC1), in turn induced by IR (79). MUC1 is a heterodimeric transmembrane glycoprotein, frequently found over-expressed in a variety of epithelial tumors and associated with proliferation, migration, angiogenesis, and chemoresistance [reviewed in (80)]. In addition to the activation of STAT3 through its phosphorylation, the over-expression of MUC1 resulted in the induction of anti-apoptotic genes downstream STAT3 such as myeloid leukaemia cell differentiation protein (MCL-1) and BCL2 like 1 (BCL-xL) (79). The major limitation of this study is the use of only one cell line system; thus, further studies are needed to understand whether this process is conserved also in other epithelial cancers and maintained among different cell lines.

**Figure 1:**
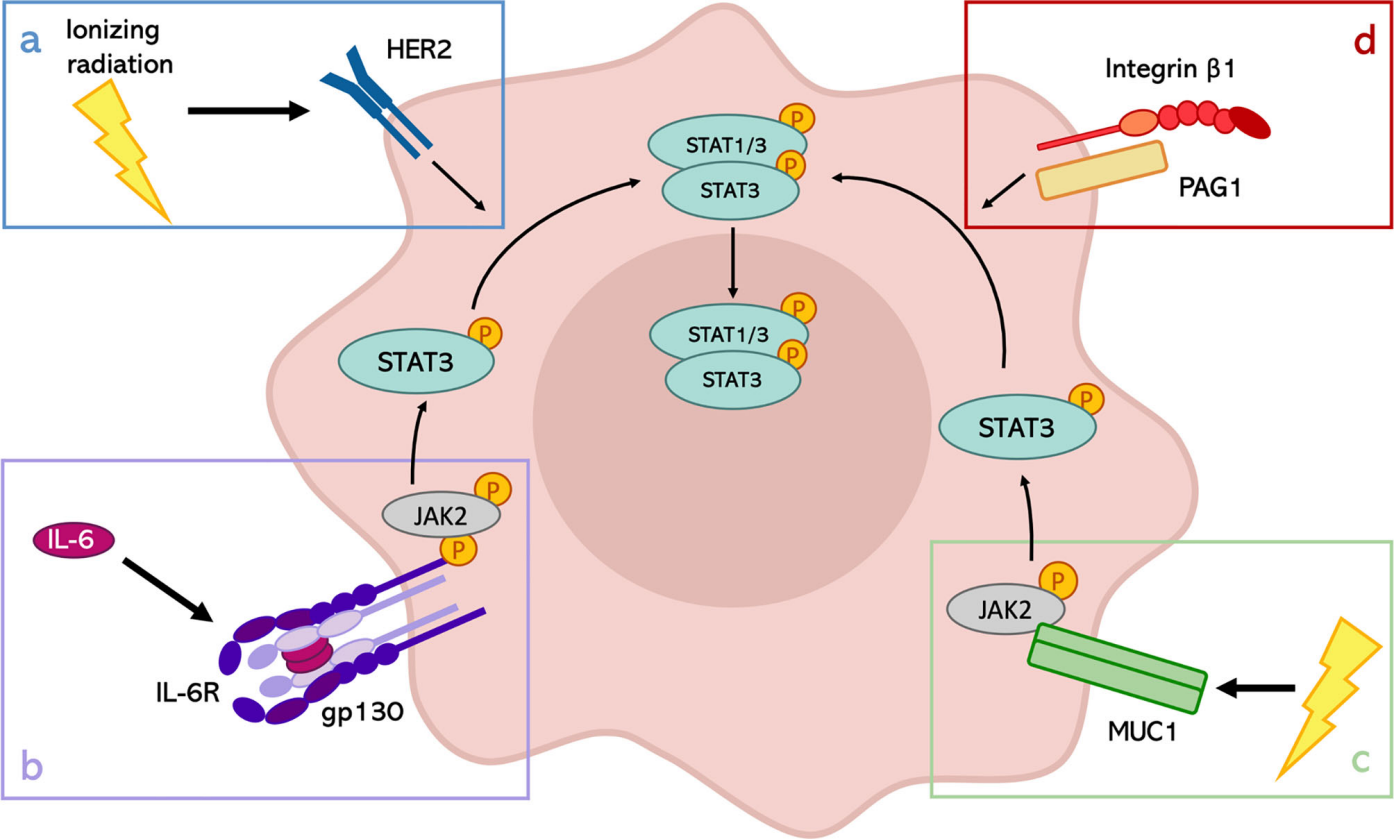
Mechanisms of STAT3 activation in response to IR. Depending on the tumor type, STAT3 can be activated in different ways after IR exposure. **(A)** STAT3 phosphorylation through the activation of HER2 receptor in HER2-positive breast cancer; **(B)** role of IL-6 signaling in the activation of the JAK2/STAT3 pathway in pharyngeal cancer, HCC and prostate cancer; **(C)** involvement of MUC1 in HCC radioresistance; **(D)** formation of PAG1-Integrin β1 complexes to induce the activation of STAT3 in laryngeal cancer.

Finally, another interesting discovery regards the formation of a membrane complex between Integrin β1 and Phosphoprotein Associated with Glycosphingolipid-enriched microdomains 1 (PAG1), which modulates the inherent radioresistance of laryngeal cancer cells (81). In particular, they demonstrated that the up-regulation of the phosphorylated tyrosine 705 of STAT3 in the radioresistant cells could be attributed to the high expression of PAG1. However, PAG1 can be considered an oncogene or a tumor suppressor gene, depending on tumor type, and its expression is not homogeneous across the different types of cancers. This observation restrains the possible applications in the clinic of this discovery but highlights the importance of STAT3 activation through different stimuli to enhance the radioresistance of cancer cells (**Figure 1**).

### Pathways Involved in STAT3-Mediated Radiation Resistance

The importance of STAT3 activation for the ability of cancer cells to acquire resistance to radiation treatment emerges considering the downstream targets. Among the canonical genes transcriptionally induced by the dimers of STAT3 there are MCL-1, BCL-xL, and, more importantly, B-cell lymphoma 2 (BCL-2). The last one is a major anti-apoptotic gene whose expression is induced by STAT3 following exposure to IR in triple-negative breast cancer cells, leading to acquired radioresistance (82).

Besides, in different types of tumors, another transcriptional target of STAT3 has emerged for its importance in conferring radioresistance to the cancer cells: Forkhead box protein M1 (FOXM1) (83). In GBM, the expression of this transcription factor is induced by the formation of a complex involving FOXM1 and the phosphorylated form of STAT3 and confers to the cells the ability to resist to radiation regulating cell cycle progression and DNA repair (84). The confirmation by the same authors that STAT3 and FOXM1 are involved in a co-regulatory positive feedback loop gives further importance to this transcription factor. The functions of FOXM1 in radiosensitivity have also been investigated in lung cancer, in which it drives the expression of kinesin family member 20A (KIF20A), augmenting cell proliferation, invasion, migration, and inhibiting apoptosis (85). The critical role of FOXM1 in mediating X-rays-induced invasion of HER2-positive breast cancer cells was pointed out also by Kambach and collaborators (86). In this case, the analysis of the downstream target genes of FOXM1 in response to IR revealed the involvement of Metalloproteinase 2 (MMP-2) and Survivin. Survivin is part of the Inhibitor of Apoptosis Proteins (IAP) family, usually expressed during fetal development but found to be re-expressed in most cancer cells. The role of this protein in modulating the sensitivity of cancer cells to radiotherapy has been described for the first time in pancreatic cancer. The correlation between Survivin expression and radiosensitivity suggested that it can be considered a constitutive and inducible radioresistance factor in pancreatic cancer cells through the reduction of Caspase-3/7 activity (87). The Caspase-dependent mechanism of action of Survivin is not the only way this factor contributes to radioresistance. Indeed, the nuclear accumulation and phosphorylation of Survivin following irradiation permit its interaction with members of the DNA double-strand breaks (DSBs) repair machinery, such as Ku70, γ-H2AX, DNA-dependent Protein Kinases, in order to sustain an enhanced DNA-DSB repair ability (66). Finally, the importance of Survivin in mediating the resistance to IR is also highlighted in breast cancer, where this protein is one of the effectors for the HER2-STAT3 anti-apoptotic function (74).

Among the targets of activated STAT3 involved in the development of resistance to radiotherapy, Lin and colleagues (72) identified SLUG. The direct binding of phosphorylated STAT3 to SLUG promoter (72) allows the increase in the expression of this zinc-finger TF, which belongs to the SNAIL family. Its siRNA-mediated down-regulation caused an increased expression of P53 Up-regulated Modulator of Apoptosis (PUMA) together with enhanced sensitivity to irradiation in cholangiocarcinoma cells (88). This observation reveals that SLUG can prompt the resistance of cancer cells to IR reducing the apoptotic process by down-regulating the expression of pro-apoptotic genes. The increase in radiosensitivity after the inhibition of SLUG expression has also been confirmed in oral squamous cell carcinoma (89), where the down-regulation of SLUG increased PUMA expression, suppressing proliferation and increasing the apoptotic rate of cancer cells.

The expression of SLUG in GBM was found to be induced by the activation of JAK/STAT3 axis in order to activate cancer cell motility and invasion (72). In this context, the STAT3-SLUG pathway mediates the radioresistance *via* enhancement of cancer stem-like properties and increased EMT-like phenotypes. These results are in line with the recent idea of the presence of CSCs, which have been shown to be involved in the resistance of some cancers to radiotherapy (14). Recently, STAT3 has been recognized as one of the mediators of radiation-induced cellular plasticity changes. Indeed, in colorectal cancer, the activation of STAT3 following irradiation allowed the cells to be intrinsically radioresistant through the activation of Cyclin D2 (CCND2) transcription (90). The direct binding of STAT3 to CCND2 promoter enhanced the expression of this Cyclin, involved in the persistent propagation of CSCs, stimulating genes involved in replication, DNA synthesis, and cell cycle progression. Moreover, the same authors demonstrated an enrichment of JAK2 and p-STAT3 in the CSCs subpopulation, together with a higher expression of CCND2, which promoted radioresistant CSCs growth.

**Figure 2:**
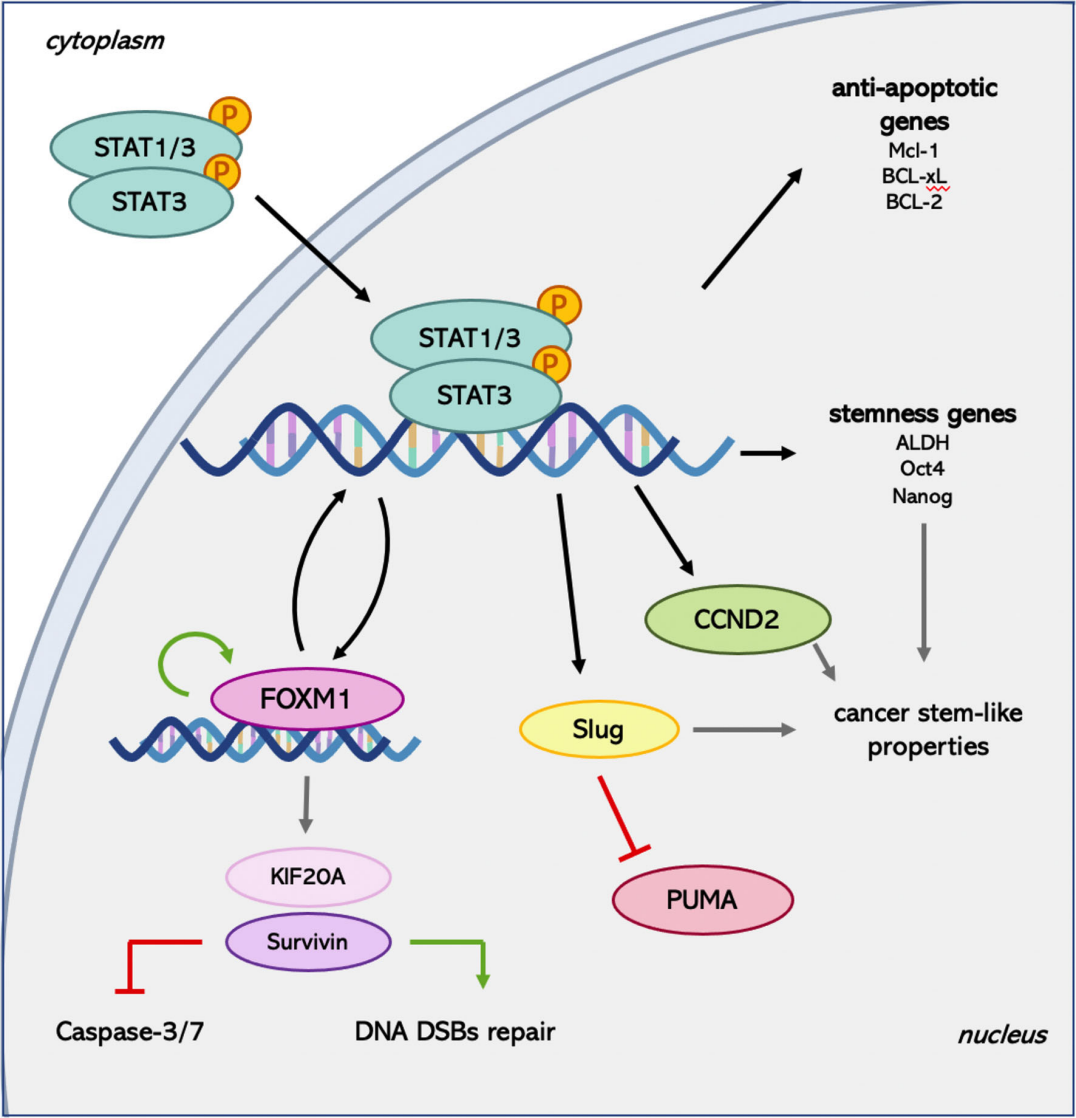
Summary of the main pathways activated by STAT3 following radiation treatment. FOXM1 is known to be able to regulate its own expression (91) and STAT3 transcription (92) establishing a positive feedback loop. Moreover, it induces Survivin expression, which can exert both Caspase-dependent and -independent downstream effects. Finally, STAT3 mediates the activation of genes involved in the maintenance of the CSCs pool, providing another system to promote radioresistance.

The role of STAT3 in the induction and maintenance of CSCs properties has also been investigated in triple-negative breast cancer. Arnold and colleagues (67) emphasized that the increase in Aldehyde Dehydrogenase (ALDH) induced by the treatment of cells with X-rays requires STAT3 activation. Furthermore, the enhanced expression of stemness genes as OCT4 and NANOG is hampered using a STAT3 inhibitor. This evidence supports the contribution of STAT3 in radiation-induced cellular plasticity, which leads to the maintenance of radioresistant CSCs (**Figure 2**).

Overall, the abundance and multiplicity of activators of the STAT3 TF and the diversity of downstream effectors induced following irradiation suggest the idea to target STAT3 as a combined treatment with X-rays to increase the efficiency of tumor eradication in the majority of cancer types. Currently, different types of STAT3 inhibitors have been developed: SH2 domain inhibitors, DNA-binding domain inhibitors, N-terminal domain inhibitors, and STAT3 antisense and siRNA [reviewed in detail in (93)]. Some of them have been already used in clinical trials for the treatment of cancer. In particular, the antisense oligonucleotide AZD9150 has entered phase I and II clinical trials for the treatment of advanced solid malignancies (94). A second promising agent that targets STAT3 and which already entered clinical trials is Napabucasin. It is a small molecule able to abrogate STAT3 signaling, for which it is possible to account 22 different clinical trials (phase I/II and III) in which it is used alone or in combination with other chemotherapeutic agents mainly to treat pancreatic, colorectal cancer and glioblastoma (94). These tumors are suitable for treatment with charged particles, given the ability of protons and carbon ions to spare the surrounding healthy tissues from high radiation doses. In addition, recently, it has been demonstrated that Napabucasin can function as a radiosensitizer for rectal cancer through the increase in ROS levels, revealing its role in the targeting of oxidative pathways and rendering it a promising agent for a combinatorial treatment with radiotherapy (95). Furthermore, an increase in STAT3 levels has been hypothesized to be a protected reaction of cancer cells to potent stressors as exposure to carbon ion irradiation (96). For this reason, a future application of one of the two most promising STAT3 inhibitors together with charged particles could be an innovative treatment option to overcome radioresistance that should be evaluated in clinic.

Despite this evidence, the inhibition of STAT3 may not be sufficient to completely eliminate the tumor following radiation treatment. As a matter of fact, the combination of STAT3 inhibitor and radiation, despite increasing the sensitivity to irradiation, could not eradicate about 10-30% of GBM cells (73). This effect is mainly due to the activation of the ERK1/2 pathway in resistant cells as a compensatory mechanism in the absence of STAT3 activation. The combination of STAT3 and ERK1/2 inhibitors causes the almost complete elimination of cells following radiation, implying that the blockage of two pathways that mediate radioresistance can be more effective in sensitizing cells to this type of treatment.

## Nuclear Factor-kappa B (NF-κB)

### Mechanisms of NF-κB Activation

**Figure 3:**
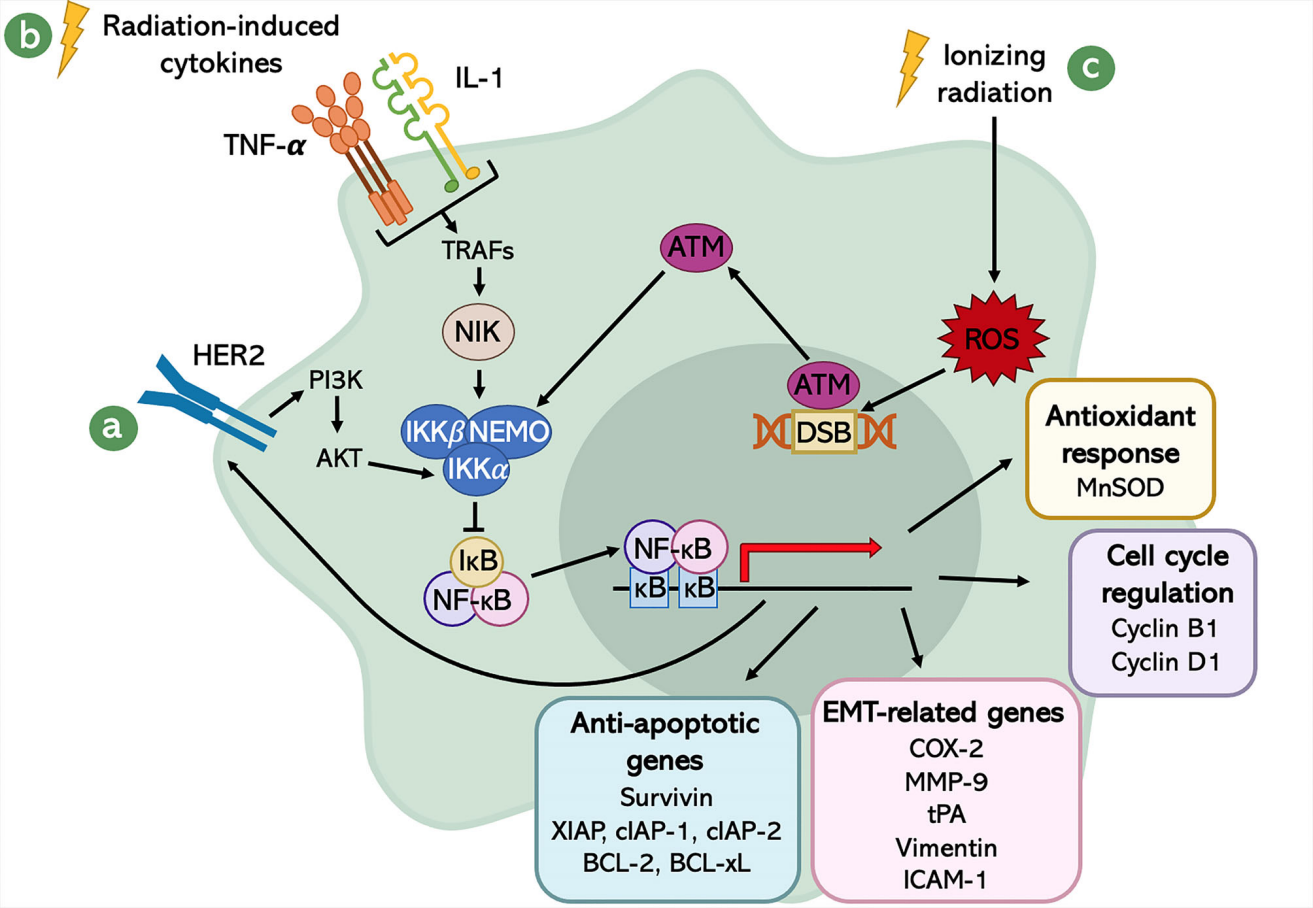
Schematic representation of the radiation-induced NF-κB signaling network. The activation of NF-κB is mediated by different stimuli: **(A)** the EGFR signaling pathway, especially through the activity of HER2; **(B)** the radiation-induced cytokines; and **(C)** DSBs generated by IR-induced ROS. The transcriptional program of NF-κB leads to several downstream events, which result in enhanced aggressiveness, increased survival and better fitness of cancer cells.

Different stimuli can lead to NF-κB activation. Since this pathway is involved in the immune system performance, it is responsive to immune modulating agents and Damage- and Pathogen-Associated Molecular Patterns (DAMPs and PAMPs). Besides that, other cellular stress factors contribute to NF-κB pathway activation: oxidative stress, cytokines, hypoxia (30), ultraviolet (UV) radiation, IR (97), and growth factors. Indeed, there is evidence that the Epidermal Growth Factor Receptor (EGFR) signaling pathway contributes to the activation of NF-κB (98). This has a strong implication in tumors, since EGFRs are remarkably up-regulated in most cancer cells. Among this family of Receptor Tyrosine Kinases (RTKs), HER2 has a central role in the sustained activity of the transcription factor NF-κB in cancer cells (99), promoting the phosphorylation and activation of IKK by AKT, one of its downstream effectors (**Figure 3A**). The relationship between HER2 and NF-κB is mutual, meaning that not only HER2 promotes NF-κB activation, but also NF-κB induces HER2 over-expression, generating a positive feedback loop (99).

Another class of molecules that plays a crucial role in the activation of NF-κB are the IR-induced cytokines, like Tumor Necrosis Factor-α (TNF-α) and Interleukin-1 (IL-1) (100). These factors are released in response to inflammation caused by IR and, upon binding with their receptor, activate a downstream cascade which involves Tumor Necrosis Factor Receptor-Associated Factor (TRAF) adaptor proteins (101). TRAFs, in turn, interact with NIK, which promotes the activation of NF-κB through the phosphorylation of the -subunit of IKK at the serine 176 (102). IKK then inhibits IκB, allowing the cytokine-mediated activation of NF-κB (**Figure 3B**).

Eventually, also DNA damage generated by X-rays radiation was shown to be involved in the up-regulation of NF-κB transcriptional activity. In fact, it is known that ionizing radiation is an exogenous source for ROS generation, which can be produced either by direct interaction with cellular targets or radiolysis of water. ROS are responsible for DSBs, which induce the activation of an important sensor of DNA damage: Ataxia Telangiectasia Mutated (ATM) serine/threonine kinase (103). This protein kinase, once activated, phosphorylates the regulatory subunit of IKK complex, NEMO, at serine 85 (104). This phosphorylation allows the nuclear export of NEMO, which positively regulates IKK that, in turn, promotes the inhibition of IκB and activates NF-κB (105) (**Figure 3C**).

### Pathways Involved in NF-κB-Mediated Radioresistance

NF-κB regulates the transcription of several genes involved in different cellular functions and processes, such as cell cycle progression, survival, protection from oxidative stress, invasion, and metastasis (26). All these properties, conferred by NF-κB activation, are linked to increased resistance to radiotherapy and chemotherapy. In particular, a well-known role of NF-κB in the process of resistance to radiotherapy is the induction of an important nuclear-encoded mitochondrial enzyme, involved in the modulation of oxidative stress: Manganese SuperOxide Dismutase (MnSOD) (106–108). The evidence of the NF-κB-modulation of the MnSOD gene, named SOD2, is given by the presence of κB sites in its regulatory regions (109, 110). This provides the proof of NF-κB-mediated responsiveness of SOD2 after various stimuli, including IR. Since the ionizing radiation-dependent toxicity is mainly caused by the production of ROS, the increased expression of this enzyme has a critical role in determining the resistance to the treatment, both in cancer cells and healthy tissues, as demonstrated in mouse skin epithelial cells (108). Despite this antioxidant enzyme is thought to have a tumor suppressor effect due to its role in avoiding malignant transformation, its function in irradiated cells is different. Indeed, in one case the effect of ROS is to promote genetic alteration leading to carcinogenesis. Instead, in the other case, ROS are responsible for IR-induced cell death. Therefore, it is clear that the circumstances in which MnSOD is over-expressed change the final outcome and the classification of this enzyme should be done in a context-dependent manner.

Another important role of NF-κB in supporting the development of adaptive resistance to radiotherapy consists of the up-regulation of the expression of several anti-apoptotic genes. Indeed, the presence of κB sites in the promoters of BCL-2 and BCL-xL has been demonstrated in different studies (111). A retrospective analysis suggests that the expression of BCL-2 is predictive for the outcome of radiotherapy in several tumor types, like prostate, laryngeal, and head and neck cancers (112–114). These observations highlight the fact that BCL-2 increased expression may be an important mechanism used by cancer cells to avoid the destructive effects of ionizing radiation. Besides, other pro-survival genes are activated by NF-κB: XIAP, c-IAP1, and c-IAP2 (115, 116). The up-regulation of these genes after radiation treatment not only protects cells from apoptosis, but also confers a growth advantage to the surviving cancer cells that will repopulate the tumor. The proof that the transcription factor NF-κB is critically involved in the promotion of cancer cell survival after ionizing radiation is also given by the fact that its inhibition makes tumor cells more prone to undergo apoptosis after DNA-damaging treatments (117). In addition, of particular interest is the NF-κB-mediated regulation of Survivin (98). Normally, Survivin is expressed in a cell cycle-dependent manner (118), but in tumors its expression is independent of the mitotic index (119). This factor is essential for the protection of cancer cells from radiotherapy- and chemotherapy-induced apoptosis, but it is also involved in the VEGF-mediated protection of endothelial cells (120), which are essential to support cancer progression through nutrient and oxygen supply.

Furthermore, the activation of NF-κB in cancer cells induces proliferation through the regulation of two critical genes involved in the cell cycle progression: Cyclin B1 and Cyclin D1 (100, 121). The latter is involved in G1 to S phase transition, and it is frequently found up-regulated in cancer cells (122). Its transcription is directly regulated by NF-κB due to the presence of κB sites in its promoter (123). Instead, Cyclin B1 controls the G2 to M phase transition and an increase in its expression is observed after an extended G2 delay due to DNA damage (124). The over-expression of Cyclin B1 is commonly found in several tumors, such as esophageal squamous cell carcinoma, laryngeal squamous cell carcinoma and colorectal cancer (125–127), and this is strictly connected to poor prognosis and the development of resistance against anti-cancer treatments, including radiotherapy. The down-regulation of Cyclin B1, using specifically designed siRNAs, has shown to increase the apoptotic rate and to decrease proliferation and colony-forming ability of several cancer cells (128), denoting the importance of this cell-cycle regulator in the promotion of carcinogenesis. Both Cyclins are involved in cell cycle arrest upon DNA damage in order to prevent the replication when the genetic material has to be repaired. As a matter of fact, this mechanism is perturbed in the vast majority of tumors, and the alteration of the cell cycle is a consolidated hallmark of cancer. The aberrant behavior of cancer cells mediated by over-expression of Cyclin B1 and D1 contributes to the accumulation of genetic lesions that will eventually result in the creation of a radioresistant and thus more aggressive tumor.

Moreover, NF-κB transcriptionally activates several genes associated with angiogenesis and metastasis, such as Matrix Metalloproteinase-9 (MMP-9), tissue Plasminogen Activator (tPA), ICAM-1, and Vimentin (30, 98, 121, 129). More importantly, also, Cyclooxygenase-2 (COX-2) is regulated by NF-κB (130). Differently from its isoform COX-1, which is constitutively expressed in almost all cells, COX-2 levels are generally undetectable in normal cells. In addition to metastasis and angiogenesis, this enzyme plays an important role in other different cancer-related processes, like neoplastic transformation, cell growth, and abrogation of the anti-tumor immune responses (131–133). The relevance of COX-2 in tumorigenesis and cancer progression is underlined by its correlation with the development of radioresistance (134, 135). Indeed, the increased expression of this enzyme upon ionizing irradiation is strictly associated with a vanishing effect of the treatment and poor prognosis in cancer patients. Furthermore, as mentioned before, another ability of COX-2 is to promote angiogenesis, inducing the secretion of several angiogenic factors, like VEGF and bFGF (136, 137). The formation of new blood vessels enhances tumor growth and, in later stages, support the process of metastasis formation together with an increased invasion potential of cancer cells.

Taking together the multiplicity of effects given by the activation of NF-κB in cancer cells, it can be concluded that this transcription factor has a critical role in determining the outcome of radiotherapy. For this reason, an increase in radiosensitivity could be achieved by combining radiation therapy with NF-κB pathway inhibitors. A well-known inhibitor of this TF is Curcumin (also known as diferuloylmethane), a derivative of the spice turmeric (*Curcuma longa*). Several *in vitro* and *in vivo* studies have been performed so far, showing the synergistic enhancement in radiosensitivity provided by Curcumin in different tumor types, such as oral squamous cell carcinoma, head and neck cancer, Burkitt’s lymphoma, colorectal cancer, hepatocellular carcinoma, and prostate cancer (138–143). In fact, the administration of Curcumin prior to radiotherapy leads to the suppression of NF-κB activation and a consequent down-regulation of NF-κB target genes, such as BCL-2, COX-2, Cyclin D1, MMP-9, and VEGF. Despite the encouraging results obtained by the previously mentioned studies, clinical trials that clearly unmask the role of Curcumin in combination with radiotherapy still lack. A limiting factor that could hinder the efficacy of this treatment modality in cancer patients is the low bioavailability and chemical instability of Curcumin (144). Indeed, obtaining a blood concentration of Curcumin comparable to the ones used in *in vitro* studies represents a great challenge that could be overcome improving the absorption through coating it in nanoparticles, liposomes or phospholipid complexes (145, 146). For what concerns nanoparticles, a phase II clinical trial in which Nanocurcumin is coupled to Radiotherapy (RT) is ongoing in prostate cancer patients (ClinicalTrials.gov number, NCT02724618), and it will hopefully provide new insights on the potential advantages given by a different Curcumin delivery.

Among the promising candidates for combination therapy with radiations there is also Sorafenib, a multiple kinase inhibitor which has been demonstrated to target MEK/ERK/NF-κB pathway, suppressing the DNA binding activity of NF-κB, both *in vitro* and *in vivo* (147, 148). Sorafenib has been already approved by FDA for the treatment of several types of cancers. Despite this, its anti-cancer efficacy as a single agent seems to be very low. Therefore, Sorafenib could be used as a pre-treatment in cancer patients to enhance the effects of radiotherapy and avoid NF-*κ*B-dependent radioresistance. So far, safety and efficacy of this treatment modality have been investigated in two clinical studies in patients with advanced hepatocellular carcinoma (149, 150). Promising results were obtained, indicating that toxicity was mostly manageable and suggesting that the administration of Sorafenib prior to radiotherapy could improve the outcome of the treatment. Moreover, a phase I clinical trial is ongoing in patients with hepatocellular carcinoma (ClinicalTrials.gov number, NCT00892658). In addition to this, another phase I study was performed to assess the safety of Sorafenib used in combination with radiation and Temozolomide (TMZ) for the treatment of high-grade glioma (151). Contrary to what was observed for hepatocellular carcinoma, in this case the administration of Sorafenib did not improve the outcome of RT and chemotherapy. The authors stated that the low effectiveness of Sorafenib was linked to its poor penetrance in the Central Nervous System (CNS). This limitation is determined by the presence of specific structures and efflux transporters in the Blood-Brain Barrier (BBB), which prevent the permeation of drugs in the CNS. Since radiations are known to enhance the permeability of the BBB (152), it would be interesting to couple Sorafenib with charged particle therapy for the treatment of gliomas. Indeed, preliminary unpublished results from our research group support the idea that proton beam irradiation with low dosages may permeabilize the BBB, improving the delivery of chemotherapeutics to the CNS. Lastly, the use of a metabolic product of estrogen, 2-Methoxyestradiol (2-ME), has shown to be effective in inhibiting NF-κB and therefore reducing the appearance of adaptive resistance in different tumor types (153–155). Unfortunately, the action of this compound seems to be tumor cell type-dependent (155), meaning that 2-ME treatment may display different effects on NF-κB activation depending on the cell type. Therefore, the choice of using 2-ME as a co-treatment with radiotherapy or chemotherapy should be evaluated very carefully. However, given the importance of NF-κB in tumor radioresistance, further studies should be carried out to completely unmask its role in inhibiting radiosensitivity in cancer cells. Moreover, a better understanding of NF-κB will be useful for the discovery of a valid negative regulator, which may work regardless the tumor cell type.

## Nuclear Factor (Erythroid-Derived-2)-Like 2 (NRF2)

### Mechanisms of NRF2 Activation

As previously mentioned, the role of NRF2 in cancer is extremely controversial. However, the association between NRF2 expression and radioresistance of cancer cells in various tumor types, as in lung cancer (156, 157), prostate cancer (158), and nasopharyngeal cancer (159), renders it a good therapeutic target to increase the sensitivity to the treatment.

Recently, the importance of NRF2 as a TF involved in the response of cells to charged particle exposure was highlighted by Hellweg and colleagues (107). In particular, they evidenced: i) how different studies had reported an involvement of NRF2 in the regulation of long-term radiation effects, ii) a dependence of radiation resistance in cancer cell lines upon NRF2 basal activity or up-regulation, iii) the relevance of this TF for the function of adult stem cells, and iv) the role of NRF2 in the cellular response to heavy ion irradiation. For this reason, we decided to focus on more recent discoveries, trying to understand the possible activators and effectors of NRF2 signaling pathway in response to IR.

One of the first articles which demonstrated that the NRF2 system is induced by X-rays as a result of oxidative stress in highly radiosensitive cells was published in 2010 (160). Indeed, in hematopoietic stem cells, the exposure to X-rays induced the transcription of a wide range of NRF2 target genes, among which there were Heme Oxygenase-1 (HO-1 or HMOX1), NAD(P)H Quinone Dehydrogenase 1 (NQO-1), and Ferritin Heavy polypeptide 1 (FTH1). Moreover, the basal mRNA levels of NQO-1 were correlated with the individual radiosensitivity of hematopoietic progenitor cells. Finally, the authors hypothesized that, upon X-rays exposure, the activation of NRF2 and the expression of its target genes can be induced directly *via* production of ROS, or indirectly through the DNA damage-mediated activation of ATM.

The increase in NRF2 mRNA and protein levels after single radiation exposure was confirmed also in tumor cells, in particular in rhabdomyosarcoma cells (161). In addition, in this tumor type, the increase in ROS levels induced after irradiation is rapidly counteracted. These results support the idea of “Redox Resetting” as a system used by malignant cells to survive in response to stresses. It is defined as the process, activated by tumor cells, to acquire a new redox balance with higher ROS levels through the up-regulation of antioxidant systems (162). Already identified as crucial mechanisms for drug resistance, the redox changes in cancer cells can be some of the factors involved in the development of resistance to IR.

Nevertheless, the induction of NRF2 can be determined not only by radiation, but also because of mutations in *KEAP1/NRF2* genes. Indeed, various alternative factors, such as point mutations, promoter methylation, and aberrant splicing of the transcript (158), could contribute to the silencing of KEAP1 protein, therefore leading to the release of NRF2 and its accumulation into the nucleus. Non-conservative amino acid substitutions in *KEAP1* gene have been reported also in lung, breast and gallbladder cancers (163–165), resulting in a non-functional protein and, consequently, in alterations in the KEAP1/NRF2 axis. The effects of KEAP1 deletion on cancer cell sensitivity have been studied in detail in lung squamous cell carcinoma. In this tumor type, the inactivation of KEAP1 results in a constitutive activation of NRF2 and decrease in ROS, promoting tumor aggressiveness, metastasis and resistance to radiotherapy and oxidative stress (166). Moreover, it has been found that the KEAP1/NRF2 mutational status can be a predictor of local recurrence after radiotherapy in patients with non-small cell lung cancer (166).

**Figure 4:**
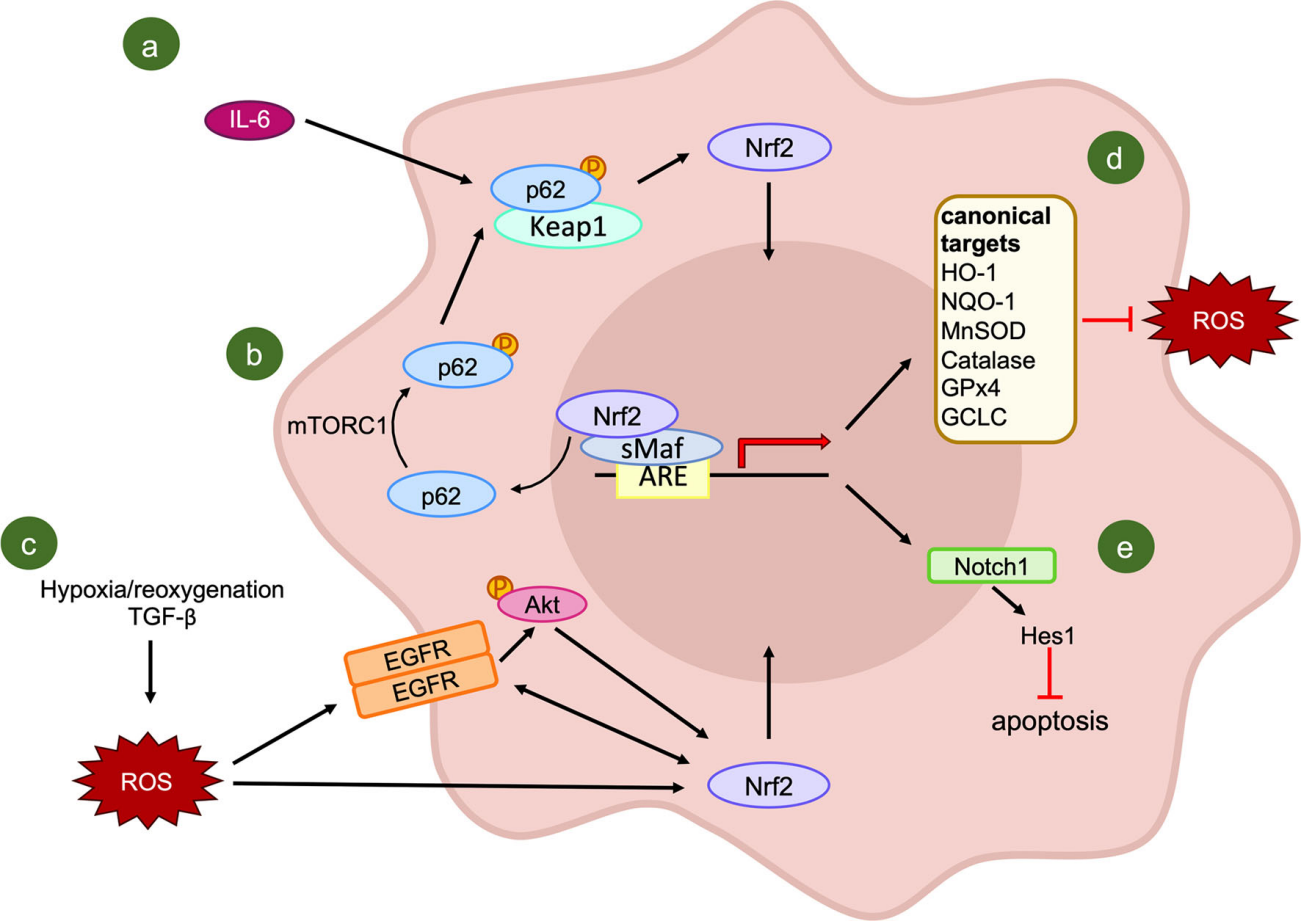
Activators and effectors of NRF2 signaling pathway following IR exposure. **(A, B)** KEAP1-dependent activation of NRF2: IL-6 induces an increase in the phosphorylated form of p62, which combines with the NRF2 inhibitor KEAP1, leading to the release and translocation of NRF2 into the nucleus **(A)**; **(B)** evidence of mTORC1 involvement in p62 phosphorylation. **(C)** A putative KEAP1-independent mechanism of NRF2 activation. **(D)** Canonical transcriptional targets of NRF2; **(E)** NRF2-mediated regulation of NOTCH1 signaling.

Considering the molecular mechanisms that can result in NRF2 activation, IL-6 has been proposed as an activator of the antioxidant pathway in oral squamous cell carcinoma due to its high expression in resistant cells (167). The blockade of IL-6 signaling through an antibody and the subsequent increase in cell radiosensitivity further confirmed the importance of this multifunctional cytokine for the survival of irradiated cancer cells. Indeed, IL-6 not only activates the downstream molecule STAT3, but also NRF2 through the increase of p62 phosphorylation. The activation of p62 permits the sequestration of KEAP1 and the release of NRF2, which induces the transcription of the ROS scavenger Mn superoxide dismutase (**Figure 4A**). Nonetheless, due to the exclusive use of *in vitro* assays, the confirmation of IL-6 implication in NRF2-axis activation needs to be verified also *in vivo*.

Currently, the inhibition of IL-6 related pathways is exploited in the clinic mainly for the treatment of rheumatoid arthritis, or other inflammatory or autoimmune diseases. Tocilizumab was one of the first monoclonal antibodies developed to block the functions of IL-6R which demonstrated increased efficacy and sufficient tolerability (168). Moreover, the use of tocilizumab, together with fenretinide and reparixin, has been applied for the treatment of oral squamous cell carcinoma, a tumor type in which radiotherapy plays an important role. The combination of IL-6 blockade with the other two drugs resulted in reduced invasion ability of CSCs enriched cultures (169).

Additionally, this monoclonal antibody was used in phase II clinical trials for the treatment of breast and pancreatic cancer (170). These results have paved the way for the development of other IL-6 inhibitors, such as sarilumab and siltuximab. Phase II clinical trials for the combination of siltuximab together with other chemotherapeutic drugs for the treatment of metastatic prostate cancer have been completed (170). To our knowledge, there is no clinical trial investigating the combination of IL-6 inhibition and radiotherapy for the treatment of radioresistant tumors. Despite this, it would be interesting to analyze the inhibition of the IL-6 axis, which could induce the activation of two of the analyzed TFs (STAT3 and NRF2), together with IR as a co-treatment approach. Indeed, the abrogation of two different ways in which cancer cells are able to acquire radioresistance could notably increase the cure rate and reduce the radiation resistance of tumors.

However, a multitude of functions has been attributed to IL-6 signaling, including a critical role in the activation of the immune system and in the mediation of the acute phase response (168). Therefore, the blockade of this cytokine could limit the effectiveness of the immune system-stimulating properties of radiotherapy.

The importance of p62 in the regulation of NRF2-mediated radioresistance of cancer cells, has been evidenced recently in tissue specimens. Starting from a significant positive correlation between phosphorylated p62 and nuclear NRF2, Wang and colleagues (171) proposed that the regulation of nuclear NRF2 by activated p62 might be the leading mechanism in the radioresistance of esophageal squamous cell carcinomas. In this tumor type, a high expression of phosphorylated p62 or NRF2 are correlated with a lower objective response rate as well as poorer progression-free survival and overall survival of patients. The association between these two factors can be explained by the evidence that, in stressed conditions, activated mammalian Target of Rapamycin Complex 1 (mTORC1) is able to induce the phosphorylation of p62 at serine 349. With this modification, p62 presents a similar domain with NRF2, it competitively combines with KEAP1 with a higher affinity compared to the unphosphorylated form, leading to the release and nuclear translocation of NRF2 (172). Moreover, activated NRF2 seems to promote the transcription of p62, forming a positive feedback loop (173) (**Figure 4B**). The major limitation of this second study is the lack of consideration of other potential confounders which can alter the results of the analysis, as the nutrition state of the patients. For this reason, further investigations on the involvement of activated p62 in the NRF2-KEAP1 axis and its role in resistance to radiotherapy are needed.

In addition to KEAP1-mediated NRF2 activation, other mechanisms result in the induction of NRF2 targets after IR exposure, without the involvement of KEAP1. As a matter of fact, in a tumor microenvironment congenial condition, obtained through the treatment of lung cancer cells with Transforming Growth Factor-beta (TGF-β) and hypoxia/reoxygenation, there is initial activation and subsequent crosstalk between Epidermal Growth Factor Receptor and NRF2 (174). This combined activation results in the resistance to IR, which is hampered by NRF2 knock-down. Furthermore, AKT signaling has been shown as the mediator of the TGF-β and hypoxia/reoxygenation-induced activation of NRF2, adding further complexity to the KEAP1-independent activation of NRF2 (**Figure 4C**).

Finally, Tian and colleagues have demonstrated an association between WNT signaling pathway and NRF2 (175). Indeed, the treatment of hepatic carcinoma cell lines with an inhibitor of Wingless/int-3A (WNT3A) increased their sensitivity to irradiation and decreased the levels of NRF2 and its target genes by limiting the entry of NRF2 into the nucleus. Even if the details of the association between these two different pathways are not known, the amplification of IR-induced effects on cancer cells due to WNT inhibitors emerges as a potential strategy to overcome resistance. Moreover, this study evidenced that the radioresistant effects of NRF2 are primarily attributable to its functions within the nucleus. This concept, together with the presence of ARE sequences in the promoter regions of NRF2 and KEAP1, suggests on one hand the ability of NRF2 to induce its own expression, and, on the other hand, the presence of an auto-regulatory feedback loop with its negative regulator.

### Pathways Involved in NRF2-Mediated Radiation Resistance

Among the genes downstream of NRF2 involved in the development of radioresistance, HO-1 appears to be induced by an increase of nuclear NRF2 in lung cancer (174) and to be down-regulated, together with NQO-1, as a result of NRF2 inhibition in hepatocellular carcinoma (175, 176) and in esophageal squamous cell carcinoma (177).

Similarly, rhabdomyosarcoma cells efficiently counteracted IR-induced increase in ROS and oxidative stress through the up-regulation of antioxidant enzymes, such as SOD-2, Catalase and Glutathione peroxidases 4 (GPX4), in a NRF2-dependent manner (161).

Surprisingly, in lung cancer, NRF2 was correlated not only with the induction of some “canonical” target genes (**Figure 4D**), such as HO-1, NOQ-1 and Glutamate-Cysteine Ligase Catalytic subunit (GCLC), but also with some “non-canonical” ones like NOTCH1 (178). Notch signaling is known to be involved in different cellular mechanisms, including cell proliferation, differentiation, angiogenesis, and tumorigenesis (179, 180). However, the regulation of Notch signaling by NRF2 in response to IR reveals a new critical player in the antioxidant response of cancer cells to radiotherapy. Indeed, after the exposure of lung cancer cells to radiation, the expression of NOTCH1 is up-regulated, while in cells knocked-down for NRF2, NOTCH1 and its downstream gene HES1 are down-regulated. This repression results in a sharp increase in protein levels of some mediators of apoptosis like BAX, cleaved Caspase-3, and Poly (ADP-ribose) polymerase-1 (PARP-1), and a decrease in the anti-apoptotic protein BCL-2 (178) (**Figure 4E**). The synergistic reduction of NRF2 and NOTCH1 signaling pathways promotes ROS accumulation and IR-induced apoptosis, suggesting a possible future strategy to overcome radioresistance in lung cancer patients.

The association between NRF2 and Notch signaling revealed to be crucial for the activation of an antioxidant program in normal conditions in the lung (181) but the induction of NOTCH1 target genes can be influenced by the model that has been used, as they do not appear to be differentially expressed in some types of lung squamous cell carcinoma (166). Therefore, a deeper investigation on the involvement of NOTCH1 in radioresistance and on its association with NRF2 in different tumor types is essential.

The inhibition of NRF2 and its antioxidant defense system confers sensitivity to radiotherapy (182). This is the reason why different studies have tried to find active compounds able to reduce its expression in order to sensitize the cells to IR. A bunch of already known drugs exhibit a radiosensitizing effect on cancer cells through different mechanisms. First of all, Genistein, used in combination with radiation, is able to increase the levels of ROS and to selectively augment the apoptotic rate in lung cancer cells through the reduction of KEAP1 promoter methylation that results in KEAP1 increased expression (183). For the same tumor type, also Brusatol has been found to be effective in the direct reduction of NRF2 in a dose-dependent manner (156).

In addition to lung cancer, also in other tumor types it is possible to achieve a radiosensitizing effect by blocking the increase in NRF2 activity: the restoration of the sensitivity to IR in nasopharyngeal carcinoma cells is associated with the inhibition of NRF2 pathways and the increase in ROS generation by Salinomycin (159). Moreover, Alpinumisoflavone and Cordycepin are two drugs with NRF2-dependent radiosensitizing properties in esophageal squamous cell carcinoma and breast cancer, respectively (177, 184). These data highlight that the modulation of the redox status of cancer cells is crucial for obtaining a radiosensitizing effect in different tumor types and that different drugs display a synergistic effect with radiotherapy. Indeed, the pre-treatment of cancer cells with NRF2 inhibitors before conventional irradiation could constitute an innovative approach to sensitize radioresistant NRF2-expressing cells to the killing action of IR. Despite being a promising opportunity to overcome radioresistance, the main limitation of this approach is the lack of knowledge of the mechanisms of inhibition and the limited specificity of NRF2 inhibitors, which are still at early phases of development (185). In this regard, we suggest the need for further studies and the development of derivatives of those molecules to reach a higher specificity and a concomitant reduction in their toxicity. The recent introduction of a modified form of Cordycepin, showing higher cytotoxicity and half-life while limiting its side effects (186), supports the continuous progress made in this field, which needs clinical evaluation for possible combinatorial treatments.

This is further supported by the finding that the already used drug Berberine promotes radiation cytotoxicity in hepatocellular carcinoma cells by enhancing oxidative stress and ROS production only in NRF2-expressing cells (176). The existence of different clinical trials combining the use of this compound with other chemotherapeutics for the control of malignancies suggests the safety of this molecule, which sets the basis for the combination of Berberine with IR in a clinical setting.

Finally, valproic acid, a histone deacetylase inhibitor used as an antiepileptic drug, contributes to the sensitization of hepatocellular carcinoma cells to proton beam radiotherapy both *in vitro* and *in vivo* (187). This effect is achieved through the enhancement of proton-induced ROS production, the abrogation of proton-mediated expression of NRF2 and HO-1 and the induction of apoptosis. In our opinion, this compound appears to be one of the most promising molecules targeting the NRF2 axis that can be used in combination with radiotherapy to decrease resistance. Indeed, two different phase 2 clinical trials have demonstrated the well-tolerated combination of valproic acid and conventional irradiation (188, 189). In the first study, the concomitant administration of the histone deacetylase inhibitor, radiotherapy, and temozolomide showed improved outcomes for the control of glioblastoma. On the other hand, in the second clinical trial valproic acid was administered in combination with radiotherapy and as a post-radiation maintenance therapy together with bevacizumab. Therefore, the future clinical combination of valproic acid with the innovative forms of charged particle therapy could constitute a breakthrough to overcome the limited effectiveness of current therapies against radioresistant tumors, such as glioblastoma.

The maintenance of the redox balance in cancer cells is one of the leading mechanisms contributing to radioresistance. However, several studies demonstrating the efficacy of the synergistic action of NRF2 inhibitors and irradiation support a new strategy to overcome this limitation. Even if the activation of this TF can vary according to the tumor type, the possibility to directly act on NRF2 stabilization and degradation or on its regulation opens new options to a common treatment. This conclusion is supported by the fact that the redox balance can contribute to stem cells self-renewal and radioresistance (190), highlighting the importance of targeting NRF2 to enhance the effectiveness of radiation therapy not only of cancer cells but also of CSCs.

## HIF-1

### Mechanisms of HIF-1 Activation

**Figure 5:**
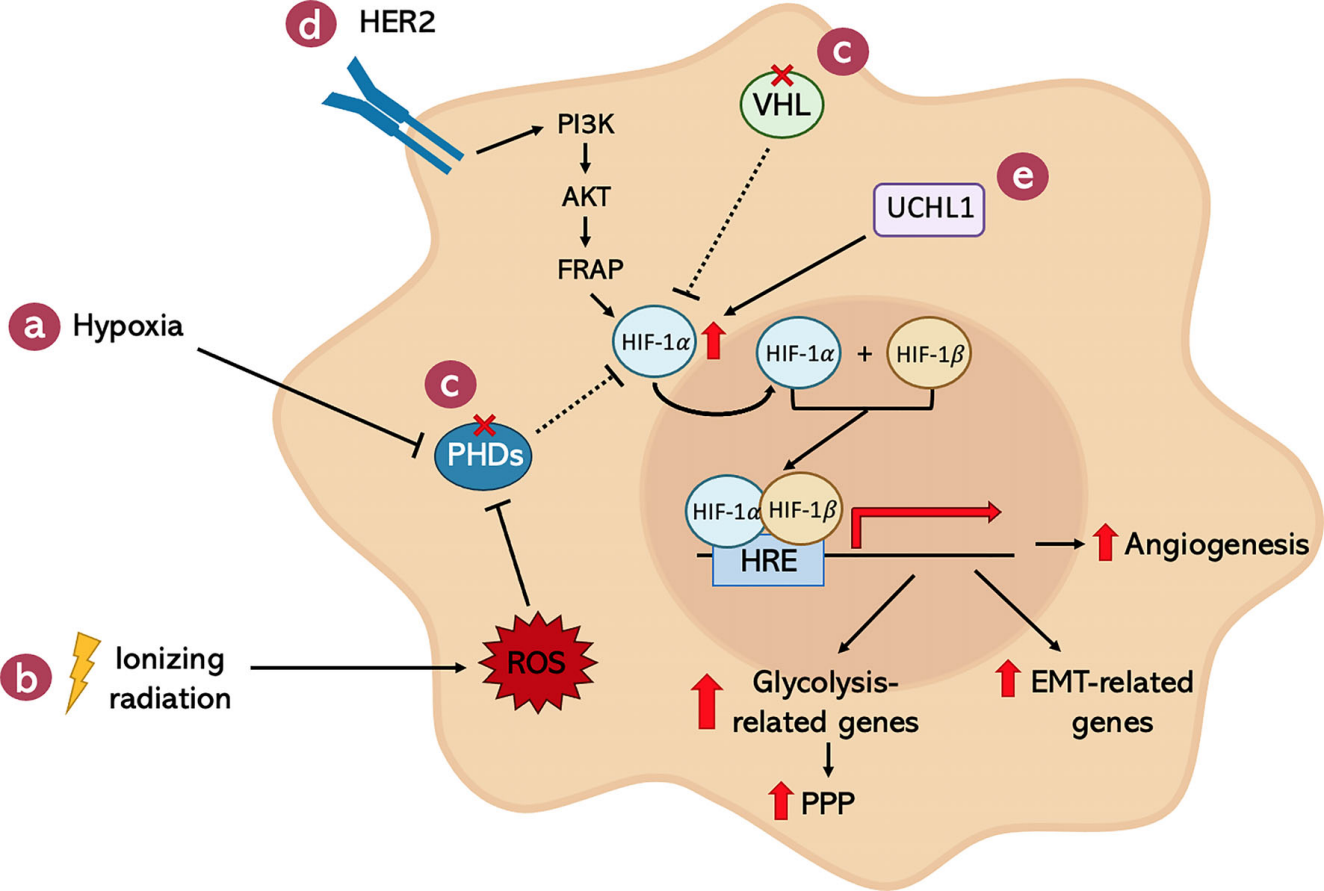
Summary of the pathways involved in the HIF-1 transcriptional activation after IR and its downstream effectors. Several clues can lead to HIF-1 activation. The most known is the presence of **(A)** a hypoxic environment, a condition frequently found in most solid tumors. Also, **(B)** ionizing radiations induce the activity of this hypoxia-related TF by the induction of ROS. Together with these external stimuli, congenital or acquired mutations promote HIF-1 activity, such as the ones found in its negative regulators: **(C)** PHDs and VHL. **(D)** Moreover, RTK receptors, like HER2, are found to induce HIF-1, which is particularly true for certain types of tumors, like breast cancers. **(E)** Lastly, HIF-1 can be activated also by the aberrant function of its deubiquitinase, UCHL1. The network established by HIF-1 transcriptional activity confers cancer cells more aggressive characteristics and protects them from the harmful effects of radiation therapy.

The hypoxic environment present in solid tumors is not the only cause of HIF-1α over-expression in cancer cells (**Figure 5**). In fact, there are several hypoxia-independent mechanisms responsible for the increase in HIF-1α activity even in the presence of oxygen. In particular, after X-ray irradiation, the huge amount of ROS generated by the treatment were found to be necessary and sufficient to activate HIF-1α (191) (**Figure 5B**). For example, high ROS levels lead to a decreased amount of Fe^2+^ through its oxidation to Fe^3+^ (192). Since Fe^2+^, together with α-ketoglutarate and O_2_, is crucial for the activity of PHDs, its reduction is responsible for the functional inactivation of these enzymes, allowing HIF-1 stabilization and transcriptional activity. In addition, also genetic alterations in genes which are critical for the negative regulation of HIF-1α have been shown to be involved in the hypoxia-independent activation of this transcription factor. Specifically, VHL-inactivating mutations are strictly correlated with the accumulation of HIF-1α protein even in the presence of oxygen (**Figure 5C**). Moreover, Receptor tyrosine Kinases (RTKs) seem to be involved in the regulation of HIF-1 through their downstream effector PI3K, AKT, and mTOR (**Figure 5D**). Indeed, there is evidence supporting the idea that, especially in breast cancer, HER2 positive tumors present enhanced HIF-1α protein synthesis (193, 194). Eventually, other studies demonstrated the relevance of deubiquitinating enzymes in the stabilization of HIF-1α. In particular, an aberrant over-expression of Ubiquitin C-terminal Hydrolase L1 (UCHL1), a known HIF-1α-deubiquitinase, corresponded to increased levels of HIF-1α in a deubiquitination activity-dependent manner (195, 196) (**Figure 5E**).

### Pathways Involved in HIF-1-Mediated Radiation Resistance

HIF-1 has been associated with radioresistance in cancer cells by activating several downstream effectors involved in different pathways linked to the repair of IR-induced damages, suppression of anti-apoptotic factors, and enhancement in the aggressiveness of cancer cells. One of the most well-known functions of the HIF-1 complex is concerning the metabolism of tumor cells. Indeed, it is well-established that malignant cells promote glycolysis and down-regulate oxidative phosphorylation (OXPHOS) in order to cope with the high anabolic demand necessary to sustain high proliferation rates. This condition is happening regardless of the presence of oxygen, and this mechanism is called Warburg effect (197). In this view, HIF-1α acts through the up-regulation of several glycolysis-related proteins: Pyruvate Dehydrogenase Kinase 1 (PDK1), which abrogates the conversion from pyruvate to acetyl-CoA; Lactate Dehydrogenase A (LDHA), which catalyzes the conversion from pyruvate to lactate; and Glucose Transporter 1 (GLUT1), involved in the uptake of glucose by the cells (198). Among these proteins, PDK1 was recently shown to be highly involved in the HIF-1α-mediated radioresistance. Indeed, the inhibition of this enzyme led to enhanced DNA damage and apoptosis, decreased cell survival, and suppressed cell proliferation after X-ray irradiation, in a way comparable to the inhibition of HIF-1α in irradiated cells (199). This observation reveals PDK1 as one of the main mediators of HIF-1α-related radioresistance. Another advantage given by the metabolic changes occurring in cancer cells is the up-regulation of glycolysis-connected subsidiary biosynthetic pathways, such as the Pentose Phosphate Pathway (PPP). As a matter of fact, PPP is involved in the production of antioxidants, starting from NADPH to obtain reduced glutathione (GSH). Of course, antioxidants are necessary for cancer cells to cope with oxidative stress caused by the high amount of ROS produced by X-ray irradiation and, therefore, contribute to the resistance observed in cancer cells after radiotherapy. The other aspect concerning the PPP is the production of ribose-5-phosphate, which is used for the de-novo-synthesis of nucleotides necessary for repairing DNA damage, thus buffering the action of irradiation.

Moreover, the most studied downstream target of HIF-1 heterodimer is the Vascular Endothelial Growth Factor (VEGF) (200), a potent signaling molecule that acts as a specific mitogen for vascular endothelial cells. This factor, together with other cytokines, is released by cancer cells, and it is involved in the promotion of tumor angiogenesis as well as permeabilization of blood vessels (201). There are pieces of evidence sustaining the idea that VEGF and basic Fibroblast Growth Factor (bFGF), another HIF-1-regulated factor, enhance the endothelial cell radioresistance (202), confirming the hypothesis that tumors actively defend themselves from IR-induced damages by protecting vessels present in the surrounding microenvironment. Indeed, the interaction between blood vessels and cancer cells is necessary both for tumor growth and metastasis formation. Logically, if malignant cells are able to move from the primary site, they will be less targetable by radiotherapy, which is a local treatment, and, in this way, they will reduce the effects of IR, leading to tumor recurrence.

Another HIF-1-driven mechanism involved in the recurrence of malignancies after radiotherapy is linked to the ability of this transcription factor to promote EMT. Indeed, HIF-1 was shown to transcriptionally up-regulate several EMT mediators such as TWIST and SNAIL (203), which are able to strongly down-regulate E-cadherin and increase the expression of mesenchymal proteins like Vimentin, VEGFRs, Fibronectin, and Matrix metalloproteinases such as MMP-2 and MMP-9. Notably, in prostate cancer, the increased SNAIL activation mediated by HIF-1 was correlated with an enhanced β-Catenin nuclear translocation (61). This event contributes to enhance the migratory and invasive potential of cancer cells, therefore promoting metastasis formation. The activated WNT/β-Catenin pathway plays a critical role in this process (204), and it is also supporting the survival of tumor cells by up-regulating anti-apoptotic and NHEJ repair proteins, minimizing the effects of radiotherapy (61). Another HIF-1-regulated EMT-driver is Plasminogen Activator Inhibitor-1 (PAI-1). This molecule has been found in cancer cells’ secretome, particularly in Non-Small Cell Lung Cancer (NSCLC) cells, and its release is radiation-induced (205). Indeed, after radiotherapy, the activation of several transcription factors, including HIF-1, leads to the secretion of this soluble molecule, allowing the communication between heterogeneous clones inside the tumor mass. PAI-1 operates through the activation of AKT and ERK1/2 pathways, thus promoting cell proliferation and survival, and through the induction of mesenchymal proteins, such as Fibronectin and Vimentin. To further promote the EMT molecular phenotype, PAI-1 is also inhibiting the expression of E-cadherin by fostering SNAIL activity (206). Given the role exerted by HIF-1 in mediating radioresistance, coupling radiotherapy with HIF-1 inhibitors could be a promising way to obtain radiosensitization in refractory tumors. Different inhibitors of HIF-1α have been developed through the years. Specifically, IDF-11774 and PX-478, (inhibiting hypoxia-dependent HIF-1 α accumulation and its translation, respectively) proved to be effective in blocking HIF-1α functions both *in vitro* and *in vivo* (207–209). Further, of particular interest is YC-1, an agent firstly developed to prevent platelet aggregation and vascular contraction and then discovered to possess a HIF-1 inhibitory function through the suppression of the PI3K/AKT/mTOR pathway and AKT/NF-κB signaling (210). Moreover, the HIF-1 inhibitory potential of YC-1 is conferred by its ability to induce HIF-1 α protein degradation (211) and to regulate the activity of this TF at a post-translational level (212). YC-1 has been demonstrated to enhance the effects of radiotherapy both *in vitro* (213, 214) and *in vivo* (202) by inhibiting HIF-1 expression and, consequently, down-regulate HIF-1 downstream genes, such as VEGF. Despite these encouraging results, YC-1 has never been tested in clinical trials, therefore further elucidation of the mechanisms of interaction between RT and YC-1 are warranted.

## Conclusions

In the last years, cancer therapy has improved a lot. Nevertheless, tumors are still one of the major leading causes of death, mainly among older people, but they unfortunately also affect younger ones.

Radiotherapy still represents one of the main therapeutic interventions for a wide range of cancer patients, resulting in beneficial effects for several of them. However, often cancer cells can survive to this aggressive treatment by the development of acquired resistance.

In this review, we have summarized the role of four major transcription factors specifically involved in resistance to radiation therapy which can lead to recurrence and treatment failure. Some of the presented effects converge on the increase in the Cancer Stem Cells’ subpopulation within the tumor. Indeed, the importance of CSCs is becoming more evident, and, therefore, these tumorigenic cells constitute an attractive target for anti-cancer therapy. Certainly, in order to better target CSCs and eliminate them, a complete understanding of these tumor-initiating cells is needed. Firstly, an adequate and practical method to identify them should be developed since the expression of CSCs markers does not simply confer a stem-cell-like phenotype and, by now, functional assays must be performed to characterize them correctly. Then, a way to specifically tackle these tumorigenic cells, together with the tumor bulk, must be achieved. For this aim, targeting the specific pathways which confer to CSCs their resistance to standard therapy may be effective in eradicating all cancer cells and thus obtaining permanent tumor control. Indeed, it is essential to distinguish between intrinsic and acquired radioresistance. The former is determined by properties that are present in these cells even before the treatment started: i) augmented DNA repair activity (i.e., stronger ATM signaling), ii) inhibition of apoptosis (increase in anti-apoptotic proteins), iii) reduced cell cycle progression (i.e., dormant CSCs), and iv) diminished ROS production (i.e., increase in scavengers). All those mentioned features are characteristic of CSCs. Instead, the latter can be activated in response to the treatment itself; the 4 TFs analyzed in this review are crucial examples for acquired radioresistance. Therefore, blocking the pathways regulated by these transcription factors could represent an essential strategy to increase radiosensitivity. Indeed, in the last years, there is growing evidence that the targeting of these TFs (at different levels: interfering with the upstream activators, the TF itself, or the downstream effectors) prior to the exposure to radiotherapy might block acquired radioresistance.

In this review, we have provided several examples demonstrating that the pharmacological inhibition of some of the analyzed TFs (i.e., NF-κB and STAT3) has proven to be effective in increasing radiosensitivity both *in vitro* and *in vivo*, and a few of them (Curcumin and Sorafenib) already reached clinical trials. All the effects summarized in this review rely on the transcriptional activities of STAT3, NF-κB, NRF2, and HIF-1. Although post-transcriptional modifications of messenger RNA (methylation, splicing, de-capping, and degradation) and post-translational modifications at protein level might change gene expression and influence resistance, the 4 TFs focus of this review regulate hundreds of genes which can confer acquired radioresistance. We, therefore, believe that the predominant impact of these regulators in radioresistance is achieved at transcriptional level. Given the importance in tumor radioresistance of the analyzed TFs previously, further studies should be carried out to completely unmask their role in inhibiting radiosensitivity in cancer cells. In conclusion, a co-treatment option through the blockage of those TFs should be considered for the majority of tumors in which there is a high rate of resistance to radiotherapy.

## Author Contributions

AB conceived this review, designed it, inserted the references, edited all other parts, and wrote the conclusions. CG wrote the CSCs, NF-κB and the HIF-1 chapters, created **Figures 3** and **5**, and contributed to the *Introduction*. CT wrote the Introduction, the STAT3, and the NRF2 chapters and created **Figures 1**, **2**, and **4**. All authors contributed to the article and approved the submitted version.

## Funding

This work was partially supported by the Starting Grant for Young Researchers, University of Trento.

## Conflict of Interest

The authors declare that the research was conducted in the absence of any commercial or financial relationships that could be construed as a potential conflict of interest.
